# A Review of the Molecular Landscape of Adenoid Cystic Carcinoma of the Lacrimal Gland

**DOI:** 10.3390/ijms241813755

**Published:** 2023-09-06

**Authors:** Sarah Kate Powell, Karina Kulakova, Susan Kennedy

**Affiliations:** 1 Research Foundation, Royal Victoria Eye and Ear Hospital, D02 XK51 Dublin, Ireland; karina.kulakova2@mail.dcu.ie (K.K.); susan.kennedy@rveeh.ie (S.K.); 2 Department of Biotechnology, Dublin City University, D09 V209 Dublin, Ireland; 3 National Ophthalmic Pathology Laboratory, D04 T6F6 Dublin, Ireland

**Keywords:** adenoid cystic carcinoma, lacrimal gland, *MYB–NFIB* translocation, *Notch*-signalling, DNA damage repair genes, epigenetics

## Abstract

Adenoid cystic carcinoma (ACC) has a worldwide incidence of three to four cases per million population. Although more cases occur in the minor and major salivary glands, it is the most common lacrimal gland malignancy. ACC has a low-grade, indolent histological appearance, but is relentlessly progressive over time and has a strong proclivity to recur and/or metastasise. Current treatment options are limited to complete surgical excision and adjuvant radiotherapy. Intra-arterial systemic therapy is a recent innovation. Recurrent/metastatic disease is common due to perineural invasion, and it is largely untreatable as it is refractory to conventional chemotherapeutic agents. Given the rarity of this tumour, the molecular mechanisms that govern disease pathogenesis are poorly understood. There is an unmet, critical need to develop effective, personalised targeted therapies for the treatment of ACC in order to reduce morbidity and mortality associated with the disease. This review details the evidence relating to the molecular underpinnings of ACC of the lacrimal gland, including the *MYB–NFIB* chromosomal translocations, *Notch*-signalling pathway aberrations, DNA damage repair gene mutations and epigenetic modifications.

## 1. Introduction

A rare, biphasic, relentlessly growing and progressing carcinoma of the secretory glands, adenoid cystic carcinoma (ACC), has a worldwide incidence of 3–4 patients per million [1,2]. ACC of the head and neck is an uncommon malignancy, accounting for approximately 1% of all head and neck cancers [1,3]. Although ACC can occur at any age, it most commonly affects patients aged between fifty and sixty years, with the sixth decade of life reported as the most common decade of disease occurrence [4]. A younger mean age at diagnosis has been reported in patients with lacrimal gland ACC (39.5 years) when compared to other anatomical sites [5]. 

Tumours of the lacrimal gland are extremely rare. A Danish study of lacrimal gland lesions from 1974 to 2007 estimated an incidence of one case per million per year. They accounted for 25% of ocular space-occupying lesions. The tumours are of epithelial origin in over 50% of cases, lymphoid in 33% and mesenchymal or metastatic cancers in the remaining 10–15% of cases [6]. Over 50% of lacrimal gland tumours are malignant. The most frequent malignant tumour in the lacrimal gland is ACC, accounting for 66% of all malignant tumours that arise in the lacrimal gland [7]. A population-based study using the Surveillance, Epidemiology and End Results (SEER) program in the USA that examined 5464 cases of ACC found that lacrimal gland ACC accounted for 1.5% (81 patients) of all ACC cases [5]. Lacrimal gland ACC is associated with poorer prognosis when compared to ACC arising from other anatomical sites [8]. This is due to its propensity for unpredictable growth rates, frequent R/M, retrograde perineural invasion (PNI) and infiltration of both soft-tissue structures and bone [9,10]. PNI is commonly observed in the absence of vascular or lymphatic invasion and is a route for tumour cell propagation [11,12]. Lacrimal gland ACC is associated with a high rate of bone invasion. Williams et al. confirmed histologically that 82% of patients had lacrimal gland fossa invasion, which indicates the need for this to be addressed during initial preoperative planning and surgical management [13]. Five-year survival rates of lacrimal gland ACC have been reported as <50%, further diminishing to 20% at 10 years [14,15].

In 1998, Font et al. analysed 12 cases of ACC of the lacrimal gland specifically and reported a 100% local recurrence rate (mean interval of 3.25 years) and a 60% mortality rate with a mean survival of 5 years [15]. Overall survival rates have been reported as up to 30% over the last decade [16]. Esmaeli et al. analysed the outcomes of 20 lacrimal gland ACC patients and noted that local recurrence occurred in 35% of cases, distant metastasis was present in 80% of patients and 65% of patients had died at the time of the study as a direct consequence of the neoplasm [17]. 

ACC at all sites has a proclivity to recur and/or metastasise [18]. Over time, distant metastasis is reported to occur in over 50% of all ACC tumours, despite initial curative surgical treatment [11,19]. Distant metastasis is related to several inciting factors, including the primary tumour site, large tumour size, age and lymph node involvement [20]. Lung is the most common site of ACC distant metastasis [20]. Long-term overall survival rates of ACC are grim and have been reported between 23 and 40%, irrespective of treatment modalities [11]. Patients with any lacrimal gland tumour will present with a palpable tumour, proptosis or eye displacement, decreased range of motion and ptosis [21]. Pain is a characteristic feature of ACC, not seen with other tumours. This is due to perineural invasion, which is an almost universal feature of ACC. This can be highlighted on MRI examination [22]. 

The current gold standard treatment option for ACC patients involves complete surgical excision with adjuvant radiotherapy (RT). To date, no effective nonsurgical systemic therapeutic agents have been identified or developed to treat ACC and/or metastatic ACC effectively. Recurrent and/or metastatic (R/M) ACC tumours are poorly characterised. Their driving molecular alterations and underpinnings remain largely unknown [23]. Metastatic ACC has an extremely poor prognosis and is largely regarded as an incurable disease generally refractive to chemotherapeutic agents [23]. Cytotoxic chemotherapy has low efficacy [2]. Clinical trials have been developed to investigate actionable therapies. A novel phase II trial investigating proton radiation or intensity-modulated RT for the improvement of local control rate and lower toxicities compared to standard RT treating salivary and lacrimal gland malignancies is ongoing [24]. RT is utilised as a local control to lower the rate of local recurrence, but it does not improve overall patient survival rates [2]. Neoadjuvant-targeted intra-arterial chemotherapy has been used recently as a systemic therapy for lacrimal gland ACC in order to improve survival outcomes, although definite improvements have not yet been shown [25]. 

ACC possesses a deceptively low-grade histological appearance and is generally characterised by indolent yet relentless disease progression. Histologically, the tumour demonstrates characteristic morphology with three distinct histological growth architectural patterns, namely tubular, cribriform and/or solid, which can be seen in various amounts in each individual tumour [26]. Tubular and cribriform patterns are associated with a low proliferative index, few mitoses and little pleomorphism. These are deceptively benign-appearing histologic features. Histologic samples may be graded based on the degree of solid growth component in their tumour. The extent of solid patterns can significantly predict patient outcomes [27,28].

**Figure 1:**
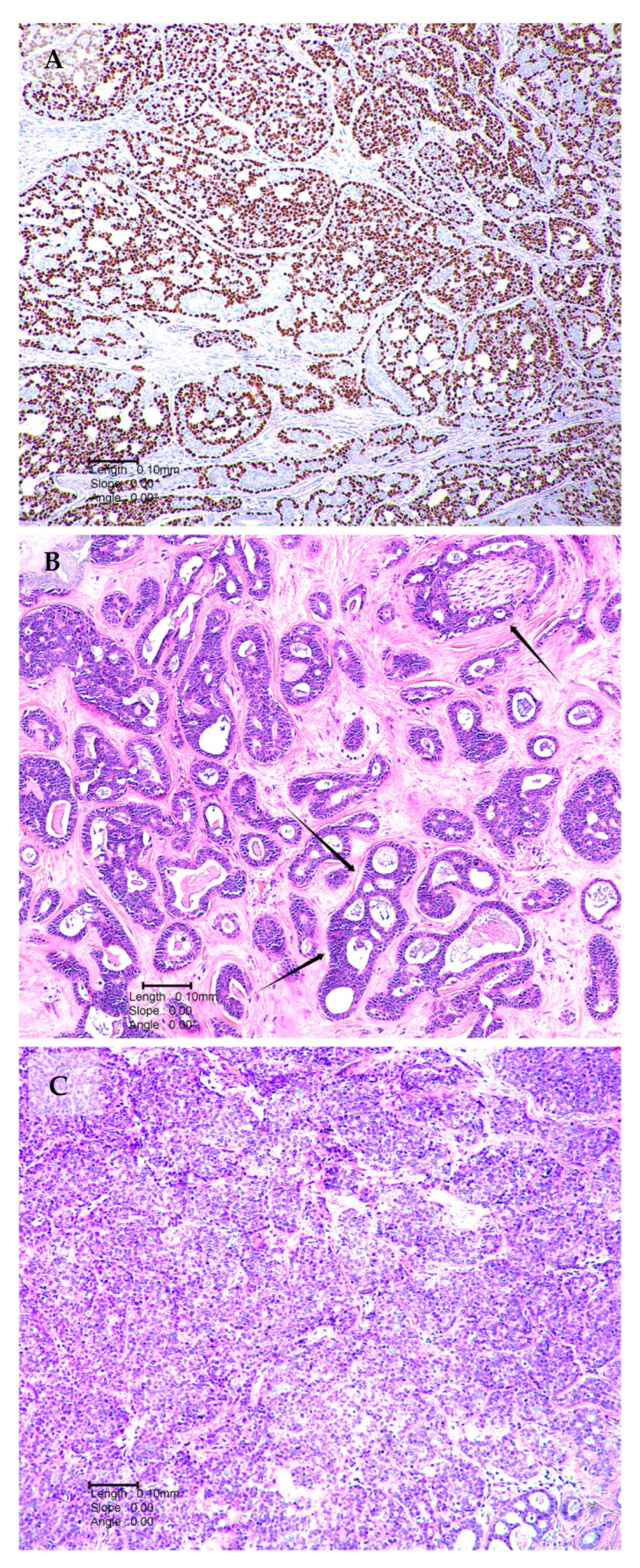
Histological patterns of ACC (**A**) p63 immunological staining demonstrating myoepithelial cells in a tumour composed of tubular and cribriform growth patterns. (**B**) Tumour with both tubular and cribriform areas. The single arrows demonstrate perineural invasion. Double arrows illustrate cribriform area. (**C**) Solid ACC growth pattern characterised the presence of pleomorphic cells, nests and sheets.

The tubular growth pattern, described in Figure 1A, is the most differentiated histological pattern. The tumour is composed of small well-formed ductal or tubular structures. This morphology has been associated with the most favourable prognosis. Tumours with this pattern alone metastasise less frequently when compared to the other patterns, and longer overall survival rates have been observed (9 years for tubular versus 8 years for cribriform and 5 years for solid [29]). Tubules are lined by inner epithelial luminal cells surrounded by myoepithelial cells with a clear cytoplasm [29]. 

The cribriform growth pattern is typical of ACC and is shown in Figure 1B. In terms of clinical prognosis and histology, it lies between tubular and solid ACC forms [30]. It is characterised by the formation of cystic structures [27]. The cribriform type has a ‘sieve-like’ or ‘cookie cutter’ appearance with luminal myxoid globules.

The solid growth pattern shown in Figure 1C is associated with the worst prognosis. It is the least-differentiated form of ACC. The cells are pleomorphic, with hyperchromatic nuclei, variable cytoplasm and a high mitotic rate, unlike the cribriform or tubular patterns. If the solid pattern accounts for >30% of the tumour, this is associated with a poorer prognosis [31]. The morphologic features are not specific to ACC and thus may represent a diagnostic challenge, particularly when attempting to differentiate ACC from other salivary gland epithelial tumours in a small biopsy or FNAC [21]. Molecular studies may be complimentary to histology and immunohistochemistry in the diagnostic setting. 

There are more than eight hundred cases of lacrimal gland ACC reported in the literature to date [21]. The cellular processes and molecular landscapes that govern the disease pathogenesis are not yet fully elucidated. This paucity of knowledge may be attributed to a lack of large-scale population-based studies, which is a direct consequence of the rarity of the tumour, thereby resulting in a scarcity of clinical cases to evaluate and analyse [32]. There is also a lack of bona fide cell lines and animal disease models available [33]. Understanding the molecular drivers of ACC will undoubtedly be critical in the development of novel therapeutic options in order to halt disease progression and metastasis. 

This review aims to explore the molecular landscape of ACC arising in the lacrimal gland and analyse established genetic mutations described in the literature that are key drivers of disease pathology, including fusion translocations, such as the *MYB–NFIB* fusion, *Notch* signalling, DNA damage response genes, and epigenetic modifications, and chromatin remodelling genes, such as *SMARCA2*, *CREBBP* and *KDM6A* [12,34]. 

## 2. *MYB/NFIB* Translocation

Genetic alterations, specifically chromosomal translocations, are hallmarks of several forms of cancer, including solid carcinomas and haematological malignancies [35,36]. Chromosomal translocations are characterised by the rearrangement of two nonhomologous chromosomes [35]. Nowell and Hungerford made the first direct link between chromosomal translocations and cancer in 1960 when they discovered the Philadelphia chromosome (translocation between chromosomes 9 and 22) and its association with the haematological malignancy, chronic myeloid leukaemia [37]. Since then, several chromosomal translocations have been associated with cancer and are often considered as the key inciting event that drives cancer pathogenesis, because their occurrence results in aberrant gene expression. 

Fusion of the proto-oncogene *MYB* with the transcription factor *NFIB* plays a major role in ACC tumorigenesis [38]. It is unique to this tumour type so that its presence is diagnostic when there is histologic uncertainty [39]. The mechanism is either through copy number gain or enhancer hijacking [18,40]. This is a pro-oncogenic phenomenon whereby the upregulation of an oncogene is driven by enhancer repositioning leading to altered chromatin interactions and ultimately increased oncogene expression [41,42]. Primary ACC tumours have relatively quiet genomes with low tumour mutational burdens [43]. A recent study by Bell et al. is the only study to date to show that the oncogenes *KRAS*, *NRAS* and *MET* are mutated in lacrimal gland ACC, thereby identifying the EGFR–RAS–RAF-signalling pathway as a potential inhibitory target [44]. 

As *MYB–NFIB* fusion is highly specific for ACC, it is considered a genomic hallmark. It is estimated that over 70% of all ACC patients possess mutations of either the *MYB* or *MYB1* genes [45]. Aberrant *MYB* gene expression is seen in approximately 60% of all ACC patients, compared to *MYBL1*, which is reported in approximately 35% of patients who are negative for the *MYB–NFIB* fusion [46]. Persson et al. were the first group to report recurrent *MYB–NFIB* fusion as a key oncogenic event in the pathogenesis of ACC, using cytogenetic and karyotype analyses of head, neck and breast ACC, as well as by reverse transcriptase polymerase chain reaction (RT-PCR). They also proposed the notion that *MYB–NFIB* fusion may be an attractive disease biomarker [47]. 

**Figure 2:**
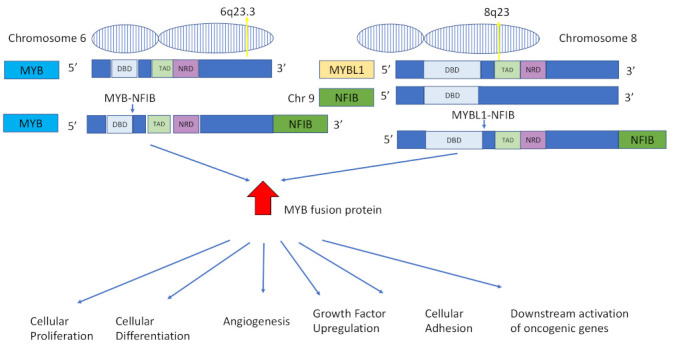
Diagrammatic representation of the *MYB/NFIB* and *MYBL1/NFIB* gene fusions. *MYB* is found at chromosomal band 6q23; *MYBL1* is found at chromosome 8q23, and *NFIB* is located at chromosomal band 9p22-23. During *MYB/NFIB* or *MYBL1/NFIB* fusion, a large part of *MYB* or *MYBL1* is fused to a small part of NFIB. The fusion results in significant upregulation of the *MYB* fusion protein, which in turn drives several oncogenic events, including increased cellular proliferation, cellular differentiation, angiogenesis, growth factor upregulation, cellular adhesion and downstream activation of oncogenic genes [36,48].

*MYB–NFIB* gene fusion most commonly occurs via the t(6;9)(q23;p23) translocation [2]. The fusion is supported by an alternative splicing system specific to *MYB–NFIB* and occurs due to *MYBL1* encoding A-MYB protein, structurally similar to *MYB*. It is currently unknown which mechanism activates *MYB* in the t(6;9) translocation event. The fusion event causes upregulation of MYB protein expression resulting in highly oncogenic *MYB* fusion protein in ACC. This upregulation is believed to be the oncogenic driver of ACC [1]. The *MYB–NFIB* and *MYBL1–NFIB* gene fusions and downstream cellular sequelae are schematically described in Figure 2. 

*MYB*, a master transcriptional activator found at chromosomal band 6q23 [36], is an oncogene essential for haematopoiesis and colonic crypt renewal, which, when overexpressed, results in increased rates of cellular proliferation, differentiation, angiogenic and growth factor upregulation, cell-cycle control, cellular adhesion and downstream activation of oncogenic genes [16,47]. *MYB* is not expressed in normal glandular cells but *MYB* mRNA and protein are highly expressed in ACC. *MYB* target genes are also overexpressed at the protein level in ACC [49]. Due to the existence of several routes for overexpression, such as selective gain at *MYB* locus [49], upregulation of the gene can be seen in both fusion-positive and a subset of fusion-negative tumours [1]. Transcription factor *NFIB* is found at the chromosomal band 9p22-23 [36]. 5′ rearrangements of *NFIB* have been described in cases of both salivary and breast ACC [46,50].

**Figure 3:**
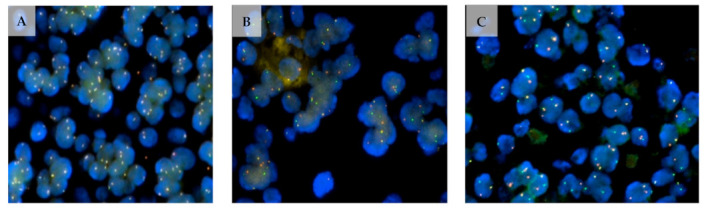
Microscopic image (magnification 1000×) of FISH analysis displaying negativity for rearrangements in *MYBL1* (**A**) and positivity for rearrangements in *MYB* (**B**) and *NFIB* (**C**). FFPE samples were cut using microtomy and stained following H&E staining protocols. The tumour location was marked off on the H&E-stained slide of each sample. For the detection of rearrangements in *MYB*, the ZytoLight SPEC *MYB* Dual Color Break Apart Probe (ZytoVision GmbH) was used. Custom-designed SureFISH *NFIB* and *MYBL1* break-apart probes were used for the detection of rearrangements of *NFIB* and *MYBL1*, respectively. The human genome (hg) build 19 was utilised for the chromosomal locations of the custom NFIB break-apart probe oligos, chr9:13740671-14140560 and chr9:14340306-14740560, and the MYBL1 break-apart probe, chr8:67076230-67474559 and chr8:67526335-68426199.

Currently, chromosomal translocations are most commonly detected using techniques, such as fluorescence in situ hybridisation (FISH) or PCR testing. Using dual-colour ‘break-apart’ probes, FISH analysis analysing *MYBL1*, *MYB* and *NFIB* rearrangements may take place [51]. In an interphase nucleus lacking translocation of the 6q23.2–q23.3 region, two orange and two green signals are expected [47]. This represents two normal, non-rearranged loci as seen in Figure 3A. Separate orange and green signals and the appearance of an orange-green fusion signal are indicative of the *MYB–NFIB* translocation and are diagnostic of ACC [47,51]. 

Targeted RNA sequencing can be used for RNA assessment and fusion gene detection, and RT-PCR and subsequent Sanger sequencing can be used to confirm novel fusions [52]. Next-generation sequencing may also be used to map chromosomal breakpoints and identify fusion transcripts and is less time-consuming than the traditional FISH analysis [53,54]. 

There are some disadvantages associated with these methods, including high failure rates when using archival formalin-fixed paraffin-embedded (FFPE) tissue. FISH may be inaccurate at delineating the exact chromosomal breakpoint [55]. Recently, McIntyre et al. conducted a pilot study that demonstrated NanoString probe technology was efficient in the detection of *MYB–NFIB* fusion. NanoString probe methodology negates the need for mRNA reverse transcription and thereby may be a more effective technique in detecting *MYB–NFIB* fusion in ACC patient samples, although more robust, large-scale studies are needed to confirm these preliminary results [56].

Pharmacological research aimed at targeting *MYB–NFIB* fusion is limited despite being widely proposed as a potential therapeutic target. This is in part due to the lack of defined ACC cell lines possessing the *MYB–NFIB* fusion [57]. Currently, a phase I single-arm multicentre clinical trial targeting fusion genes as a potential treatment through the use of a TetMYB vaccine and antiprogrammed death 1 (PD1) antibody is ongoing [58]. If the vaccine is found effective, patients with *MYB* overexpression and *MYB–NFIB* fusions in both cohorts will be eligible. A recent study identified that *MYB–NFIB* fusion is regulated by AKT-dependent insulin-like growth factor receptor 1 (*IGFR1*) signalling, which also has ramifications in terms of potential treatment modalities aimed at inhibiting *IGF1R/AKT* signalling [33]. 

### 2.1. MYB–NFIB Fusion in Salivary Gland ACC

Mitani et al. studied the prevalence of *MYB–NFIB* fusion in 123 patients with salivary ACC. They reported that the fusion was present in 28% of patients with primary ACC and 35% of patients with metastatic ACC. Importantly, the *MYB–NFIB* fusion was not detected in other salivary carcinomas, highlighting its specificity for ACC and its potential as a clinical diagnostic biomarker [46]. Fujii et al. evaluated the presence of *MYB*, *MYBL1* and *NFIB* in 33 patients with ACC of the salivary gland using FISH analysis. They noted that 88% of patients had aberrant *MYB*, *MYB1* or *NFIB* expression. Furthermore, patients with *MYC* overexpression had significantly shorter disease-free periods, indicating that *MYC* overexpression may have a role as a prognostic biomarker [45]. 

Ferararotto et al. performed a proteogenomic analysis on 38 ACC tumour specimens. They found two ACC molecular subtypes, namely ACC-I and ACC-II. *MYB* and Notch-activating genes were overexpressed in ACC-1 samples, whereas aberrant *TP63* and receptor tyrosine kinases (*AXL* and *EGFR*) RNA and protein expression were evident in ACC-II specimens. The proto-oncogene *BCL-2* was also upregulated in ACC-1, and its inhibition may be an attractive therapeutic target. These results may have important implications in terms of developing personalised therapeutic targets for each ACC subtype [59].

Using bulk RNA sequencing analysis, Brayer et al. analysed samples from 56 salivary gland ACC patients and subdivided ACC tumours into three distinct cohorts on the basis of their genetic expression profiles [60]. Group 1 (76% of patients) had a median survival of >10 years post-surgical intervention and were characterised by the presence of *MYB* or *MYB1* differential gene expression. Group 2, known as the ‘no *MYB* group’ (10% of patients), did not express either *MYB* or *MY1B* on RNA-seq analyses but had similar survival rates to the main group. Group 3 (14% of patients) had a significantly poorer prognosis when compared to the other two groups. TP63 was significantly underexpressed in this patient group [60]. Frerich et analysed 68 ACC tumour samples using RNA sequencing [61]. *SOX4* and *EN1* gene overexpression were closely linked with the upregulation of *MYB* or *MYBL1*, indicating they may behave as *Myb*-directed targets. A total of 80% of the patients in this study had *MYB* or *MYBL1* expression, and very different differential gene expression analysis was observed in these patients when compared to patients expressing neither *MYB* or *MYBL1*. This study also identified a subgroup of patients who had poor survival outcomes. *SOX4* and *CTNNB1* were overexpressed in this patient cohort, and *PIK3R1* and *TP63* were under expressed [49]. Further research is necessary to stratify ACC patients at diagnosis according to their gene expression signatures and to identify those requiring prompt, aggressive treatment [49].

### 2.2. MYB–NFIB Fusion in Lacrimal Gland ACC

There is limited literature describing *MYB–NFIB* fusion in lacrimal gland ACC. Chen et al. performed a retrospective review of cases of lacrimal gland ACC at the Mayo Clinic, which comprised 12 cases over 25 years. Using FISH analysis, they noted that *MYB* rearrangement was present in 58% of cases, but its presence had no significant impact on survival rates [26].

Von Holstein et al. analysed 14 cases of lacrimal gland ACC and found that 50% of patients expressed *MYB–NFIB* fusion. Lacrimal gland ACC and salivary gland ACC are genetically and clinically similar [62]. The *MYBL1–NFIB* gene fusion has been described in salivary gland ACC and breast ACC, but as of yet, there are no reports of *MYBL1* rearrangements in ACC of the lacrimal gland [50,63].

Larger-scale studies looking at the genetic profile of lacrimal gland ACC are necessary in order to determine whether lacrimal gland ACC can also be subdivided into groups based on their genetic signature, thereby paving the way for precision medicine.

## 3. Notch-Signalling Pathway

**Figure 4:**
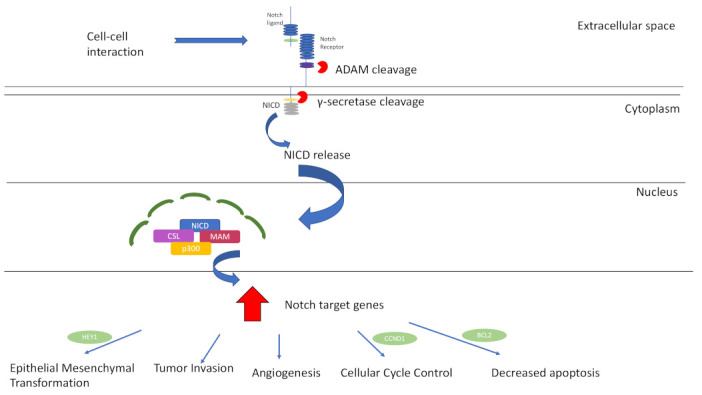
The *Notch*-signalling pathway and downstream cellular sequelae in ACC. *Notch* receptors are bound to *Notch* ligands at epidermal growth factor repeats, resulting in a conformational change. *Notch* undergoes two cleavages: firstly by ADAM metalloproteases and again by γ secretase. *Notch* intracellular domain (NICD) is then released from the cytoplasm and travels to the nucleus where it interacts with various transcriptional coactivators, including DNA-binding protein CFB1/Suppressor of Hairless/LAG1, mastermind (MAM) and p300. These interactions result in upregulation of target genes, having downstream cellular consequences, including epithelial–mesenchymal transformation (EMT) (via HEY1), tumour invasion, angiogenesis, regulation of cell-cycle control (via CCND1) and apoptosis (via BCL2) [48,69,70,71].

The *Notch* intracellular-signalling pathway is a highly conserved pathway that plays an integral role in embryogenesis. It is also a key regulator of several cellular processes in adults, such as proliferation, differentiation and survival [64]. The *Notch*-signalling pathway is described in Figure 4. Dysregulated *Notch*-signalling pathway has been implicated in cancer pathogenesis for decades, since its first association with human T-cell acute lymphoblastic leukaemia (ALL) was noted in 1991 [65]. Aberrant *Notch* expression is associated with many solid carcinomas, including breast, prostate, colorectal, lung and central nervous system cancer [66]. In terms of its tumorigenic properties, *Notch* upregulation promotes epithelial-to-mesenchymal transition (EMT), angiogenesis, tumour invasion and cellular survival [67]. Depending on the context, *Notch* has tumour-suppressive genetic (TSG) properties [43]. *Notch* behaves as a TSG in oral squamous cell carcinoma [68].

In recent years, several bodies of work have aimed to uncover the association with aberrant *Notch* signalling and ACC disease pathogenesis. *Notch* pathway mutations have been shown to be present in between 11 and 29% of ACC patients [12,72]. Aberrant *Notch* signalling promotes ACC proliferation and plays an important role in tumour metastasis via neoangiogenic induction, promoting tumour growth and survival [73]. *Notch1*, *Notch2*, *Notch3* and *Notch4* were all upregulated in metastatic salivary gland ACC cell lines. Furthermore, an important role for *Notch4* was identified in the metastasis of salivary gland ACC, and the knockdown of *Notch4* using small-interfering RNA (siRNA) inhibited metastatic invasion of ACC cells [74]. Ho et al. analysed 1045 ACC patients and found that *Notch* was significantly overexpressed in R/M tumours when compared to primary tumours [43]. Another study by Su et al. demonstrated that *Notch1* was overexpressed in salivary gland ACC R/M tissue samples, and this overexpression was associated with disease R/M [75]. They showed that *Notch1* overexpression drove cellular processes, such as tumour migration, invasion, proliferation and cell growth. Inhibition of Notch1 decreased tumorigenicity through promoting programmed cell death. These results further demonstrate that *Notch1* plays an integral role in ACC metastasis [75]. *Notch1* activation promotes upregulation of the antiapoptotic and cell-cycle genes *BCL-2* ad *CCND1*, which is a potential mechanism by which *Notch1* drives ACC metastasis [73].

Ferrarotto et al. genotyped 102 ACC tissue samples and demonstrated that *Notch1* mutations were associated with an aggressive subgroup and conferred a poor prognosis [76]. *Notch1* mutations were associated with bone and liver metastasis as well as a significantly shorter overall survival when compared to the *Notch1* wildtype cohort. This study also suggests that further research investigating *Notch1* inhibitors as potential therapeutic targets in the nonsurgical management of ACC is warranted [76]. Sajed et al. performed whole-exome sequencing on 194 cases of confirmed ACC of the salivary gland and demonstrated that *Notch1* gain-of-function mutations correlated with a solid disease histological pattern, as well as being associated with a poorer clinical outcome [77]. Rettig et al. analysed whole-genome sequencing results from 25 ACC patients and found that *Notch1* was the most commonly mutated gene, present in 12% of tumours [78].

There is a growing body of evidence that *Notch1* inhibition may be a promising treatment option for ACC. A phase I clinical trial found that 17% of patients demonstrated at least partial disease stabilization when treated with the *Notch1* inhibitor, brontictuzumab [79]. Preliminary results involving the upstream *Notch1* inhibitor CB-103 showed that the median progression-free survival was almost 22 weeks, and the disease control rate was 79% after 8 weeks and 58% after 20 weeks. A total of 16% of patients with *Notch* mutations had stable disease confirmed by radiology after six months [80]. A study of AL101 in patients with ACC (ACCURACY) is a phase II multicentre trial analysing the effect of *Notch* inhibitor AL101 in R/M ACC patients harbouring *Notch1*-, *Notch2*-, *Notch3*- *or Notch4*-activating mutations. AL101 is a selective gamma-secretase inhibitor, and preliminary trial results indicate a disease control rate of 68%, with 15% of patients demonstrating partial responses [81].

### Notch Signalling and Lacrimal Gland ACC

*Notch* signalling is essential for the correct development of the lacrimal gland [82]. *Notch* has recently been identified as a player in the pathogenesis of lacrimal gland ACC, although there are limited studies in the literature that fully establish its role. Sant et al. used whole-exome sequencing to screen for and identify mutations in lacrimal gland ACC. *Notch1* and *Notch2* mutations were associated with functionally severe disease. These mutations were present in 31% of patient samples analysed. This study identifies the *Notch* signalling pathway as a potential therapeutic target for lacrimal gland ACC [83]. Anjum et al. studied the expression of *Notch1* receptor for the first time in the lacrimal gland, and activated *Notch 1* (NICD) in 23 cases of lacrimal gland ACC. They found that *Notch1* receptor was overexpressed in 65% of patients and NICD was overexpressed in 39% of cases. *Notch1* overexpression conferred a poorer prognosis and was correlated with significantly reduced disease-free survival rates [9]. This study is the first to identify a key role for *Notch1* in lacrimal gland ACC pathogenesis, and its overexpression is associated with an aggressive disease phenotype. *Notch1* is overexpressed in other types of cancer, including papillary thyroid carcinoma [84], colorectal cancer [85], lung adenocarcinoma [86] and breast cancer, and its upregulation is associated with aggressive disease phenotypes, disease progression [66] and poor clinical prognoses.

Overall, the collective deregulation of *Notch* signalling pathway genes consolidates it as a central mediator of ACC pathogenesis.

## 4. DNA Damage Repair Gene (DDRG) Mutations

DNA damage repair genes (DDRGs) are integral for the proper maintenance of genomic stability [87,88]. There are two primary DDR pathways—base excision repair and nucleotide excision repair [89]. Collectively, DDR response pathways comprise a complex network of proteins that identify DNA damage, signal its presence and promote its repair in a substrate-dependent manner [90]. Approximately 450 genes code for DDR proteins [91]. DDRGs are important cell-cycle regulators, as well as critical initiators of apoptosis in situations when DNA is damaged beyond repair.

Dysregulation and aberrant expression of DDRGs have been implicated with the development of a plethora of disease pathologies, including neurodegenerative conditions, ageing, immune deficiencies and cancer pathogenesis [89]. Defective DDRGs lead to a blockade of critical metabolic processes, such as transcription and replication, or mutations resulting in cellular senescence and apoptosis [90,92]. DDRG mutations promote carcinogenesis and accelerate cancer progression. DDR pathways have also been studied as potential targeted therapeutic options in cancer treatment [91,93].

ACC occurs due to somatic aberrations in the human genome. Genetic alterations are defined by high-level amplifications, somatic mutations, structural variants and homozygous deletions. These alterations take place at a cellular level in somatic tissues post-fertilisation and do not involve the germline; rather, these mutations spontaneously occur throughout a patient’s lifetime, commonly as a result of mistakes in DDR mechanisms [90]. Due to the low mutational burden observed in common tumour suppressor genes and oncogenes, a predisposition is present whereby alterations in specific transcriptional regulatory genes are upregulated by alterations in chromatin structure, which are the driving force of the neoplastic process in ACC [94,95].

Although there is a lack of data in general exploring the role of DDR pathways in salivary gland malignancies [96], DDR genes have been recently implicated in ACC pathogenesis. Ho et al. demonstrated that 27% of ACC patients demonstrated altered expression of DDR/checkpoint signalling genes, including *TP53*, *UHRF1*, *ATM* and *BRCA1* [12]. Andersson et al. were the first group to note the overexpression of *ATR* in primary ACC cell lines. *ATR* is a DNA-damage sensor kinase that is activated by replication stress and acts downstream of *MYB* [18]. On a cellular level, *ATR* overexpression exhibits oncogenic properties and is critical for tumour survival with aberrant cell-cycle check points or DNA repair mechanisms [97]. Lecona et al. demonstrated that *ATR* overactivation resulted in the activation of DDR genes, cell-cycle genes and DNA replication genes. The net effect is genomic stability, which in turn promotes cancer pathogenesis. Furthermore, treatment with an *ATR* kinase inhibitor (VX-970) induced programmed cell death in ACC cells that are *MYB*-positive and also significantly decreased cellular proliferation rates and inhibited ACC tumour growth, with one mouse demonstrating tumour regression. This study is the first to identify *ATR* as an attractive novel downstream therapeutic target of *MYB* [98]. There are several clinical trials ongoing targeting the development of *ATR* inhibitors for several cancer types [98].

Felix et al. examined the mutational profile of DDR genes in salivary gland ACC pathogenesis and noted high expression levels of a nucleotide excision repair gene *XPF* in minor salivary gland ACC samples [96]. Furthermore, the APE1 protein that acts as a redox transcription factor coactivator and is involved in the base excision repair pathway was expressed in the nucleus/cytoplasm of 50% of ACC cases. Cystoplasmic/nuclear-cytoplasmic *APE1* expression is associated with increased cellular proliferation and metabolism [96,99].

*ARID1A* is one of the most frequently mutated genes in cancers. It is involved in transcription inactivation and repression, similar to *SMARCA2*, a TSG found mutated in 5% of ACC patients [39]. Alterations in the gene in conjunction with other ACC driver mutations lead to ACC proliferation due to its inactivating effect resulting in a TSG function. Inactivation of *ARID1A* can lead to synthetic lethality via inactivation of the SWI/SNF complex by deletions or knockdowns compromising double-stranded DNA repair. This increases sensitivity to DNA-damaging agents [100]. Loss of *ARID1A* impairs the G2/M DNA damage checkpoint.

Taken together, disruptions in the DDR pathway increase mutagenesis and genomic instability thereby promoting neoplastic progression [92].

## 5. Epigenetic Modifications in ACC Pathogenesis

The field of epigenetics refers to the heritable modification of gene expression without alteration of the DNA sequence itself [101]. Epigenetic modifications have been widely studied in tumorigenesis, and it is known that malignant cells exploit epigenetic mechanisms in order to control invasion, therapy resistance and growth [102]. Epigenetic alterations involve chromatin modifications in response to hormonal and/or environmental stimuli, as well as histone acetylation affecting chromatin stability and noncoding RNA regulations [103]. Targeting epigenetics is a promising therapeutic strategy for cancer treatment, either as a monotherapy or in conjunction with adjuvant treatment [103].

In terms of ACC pathogenesis, epigenetic modifications remain poorly understood, but have shown to upregulate tumorigenic pathways associated with poor prognosis, such as MYB translocations with NFIB. Using exome sequencing of 24 patients with confirmed ACC, Frierson et al. identified chromatin remodelling mutations in 50% of ACC patients, including *ARID1A*, *CREBBP*, *EP300* and *KDM6A*. Aberrant expression of these chromatin remodelling genes may imply that ACC pathogenesis is driven by histone modifications, resulting in transcriptional reprogramming [94]. Mutations in the epigenetic modifier genes *CREPPB*, *KANSL1* are involved in histone acetyltransferase activity, KDM6A involved in histone demethylase activity, BCOR involved in histone deacetylase activity and ARID1B in the SWJ/SNF chromatin remodelling complexes are found in approximately 50% of ACC. Recurrent/metastatic ACC are enriched for BCOR and KDM6A mutations [104].

Using whole-genome sequencing analyses of 25 fresh-frozen salivary gland ACC samples, Rettig et al. proposed a role for epigenetic regulation in ACC pathogenesis. Their results noted alterations in the chromatin-remodelling genes *SMARCA2*, *KDM6A* and *CREBBP* [78]. Ho et al. also demonstrated that 7% of ACC patients possessed a C*REBBP* mutation, a histone acetyltransferase enabling transcription across critical signalling pathways, had been mutated in 7% of patients in previous ACC studies [12]. TERT-promoter genes have been described in ACC tumours without *MYB/MYBL1* fusions and without *Notch* mutations, possibly representing an alternative method of tumourigenesis [43]. Further studies are necessary to fully elucidate the role of chromatin remodelling and epigenetic modifications in ACC pathogenesis. They could be targeted by epigenetic drug therapies.

## 6. Future Directions and Conclusions

ACC remains both a diagnostic and treatment challenge for clinicians, and the molecular mechanisms that underscore disease pathogenesis are only beginning to emerge. This review aimed to explore the molecular landscape of ACC pathogenesis, paying particular emphasis to *MYB/NFIB* gene fusion and its potential as a diagnostic biomarker, as well as reporting on the role of aberrant *Notch* signalling as prognostic markers, mutated DNA damage repair genes and epigenetic modifications as drivers of disease pathogenesis. Whilst clinical trials aimed at inhibiting *Notch* have shown initial promise, it is of paramount importance that further large-scale studies aimed at targeting the underlying cellular mechanisms and biological processes that govern ACC are conducted. The development of targeted therapeutic options to ameliorate disease will pave the way for personalised ACC therapy, ultimately improving overall patient survival rates and reducing morbidity and mortality associated with the disease.

## Data Availability

No new data were created or analyzed in this study. Data sharing is not applicable to this article..
